# Acute interstitial nephritis induced by *Dioscorea quinqueloba*

**DOI:** 10.1186/1471-2369-15-143

**Published:** 2014-09-03

**Authors:** Ha Yeon Kim, Sung Sun Kim, Soo Hyeon Bae, Eun Hui Bae, Seong Kwon Ma, Soo Wan Kim

**Affiliations:** 1 Department of Internal Medicine, Chonnam National University Medical School, 42 Jebongro, Gwangju 501-757, Korea; 2 Department of Pathology, Chonnam National University Medical School, Gwangju, Korea; 3 Department of Dermatology, Chonnam National University Medical School, Gwangju, Korea

**Keywords:** *Dioscorea quinqueloba*, Acute interstitial nephritis

## Abstract

**Background:**

The use of herbal medicine may be a risk factor for the development of kidney injury, as it has been reported to cause various renal syndromes. *Dioscorea quinqueloba* is a medicinal herb that is used as an alternative therapy for cardiovascular disease and various medical conditions.

**Case presentation:**

A 52-year-old man was admitted with complaints of skin rash and burning sensation. He had ingested a raw extract of *D. quinqueloba* as a traditional remedy. Laboratory tests revealed the following values: absolute eosinophil count, 900/mm^3^; serum creatinine level, 2.7 mg/dL; and blood urea nitrogen, 33.0 mg/dL. The immunoglobulin E level was markedly increased at 1320.0 IU/mL. Urinalysis revealed a fractional excretion of sodium of 3.77%, protein 1+, and blood 3+. Histological examination of the renal biopsy specimen showed a diffusely edematous interstitium with infiltrates composed of eosinophils, lymphocytes, and neutrophils.

**Conclusion:**

Here, we present the first reported case of biopsy-proven acute interstitial nephritis following ingestion of *D. quinqueloba* associated with skin rash, eosinophilia, and increased plasma immunoglobulin E level.

## Background

Acute interstitial nephritis (AIN) is an important cause of reversible acute kidney injury [1], which is pathologically characterized by an inflammatory infiltrate in the renal interstitium [2]. The incidence of AIN ranges from 2–3% in native renal biopsies to 10–15% in cases of acute kidney injury (AKI) [3]. Although the pathogenesis of AIN is not fully understood, an immune-mediated mechanism has been postulated [4]. Furthermore, some medications and infections have been reported to trigger such pathologic immune reactions.

The use of traditional herbal medicine is common worldwide. Herbal medication is distributed through various channels without guarantee of reliability. Furthermore, various other remedies, healthy food alternatives, and complementary therapies are used indiscriminately. However, as with the use of conventional medicine, complications causing severe illness may arise in some patients. Since the kidney is a major organ for the excretion of exogenous compounds, AKI is one of the frequent complications. Although the precise mechanisms underlying the toxicity of traditional herbal medicines are unknown or have not yet been investigated, the increasing clinical evidence is contributing to a better understanding of the pathophysiology related to their use.

To diagnose AKI related to the use of herbal medicines, a thorough clinical interview is necessary. However, in practice, the diagnosis of drug-induced kidney injury is difficult in most cases, because most patients believe that herbal medicines are not “true” medicines. Furthermore, a kidney biopsy and laboratory analysis of the components of the herbal medicine need to be ordered to confirm a suspected diagnosis based on the presence of the toxin. However, the availability of biopsy and laboratory analysis findings in patients with drug-induced kidney injury is limited. Recently, the authors reported 2 cases of AKI associated with *Dioscorea quinqueloba* ingestion [5], in which the pathogenesis remains unclear. Here, we present a case of biopsy-proven AIN following ingestion of *D. quinqueloba*, which was associated with skin rash, eosinophilia, and increased plasma immunoglobulin (Ig) E level.

## Case presentation

**Figure 1:**
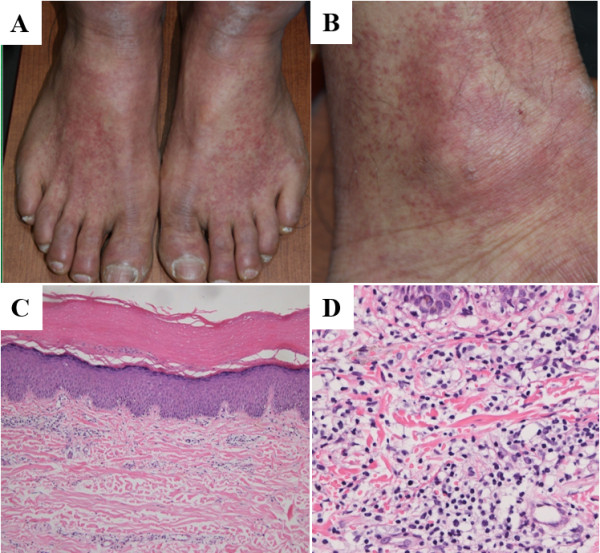
**Gross and microscopic exam of the skin. A&B** Erythematous, variable-sized macules and patches on **(A)** both the dorsum of the feet and **(B)** ankle area. **C** Superficial dermal perivascular chronic inflammatory cell infiltrate (×100, H&E stain). **D** Slight interface change and predominant lymphohistiocytic infiltrate in the superficial dermal and perivascular area (×400, H&E stain).

**Figure 2:**
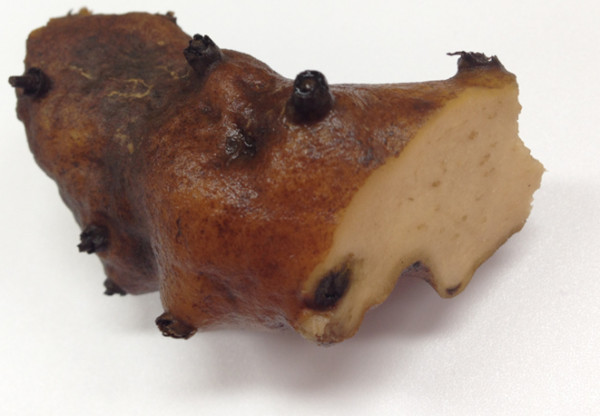
**A sample of *Dioscorea quinqueloba *** **ingested by the patient.**

**Figure 3:**
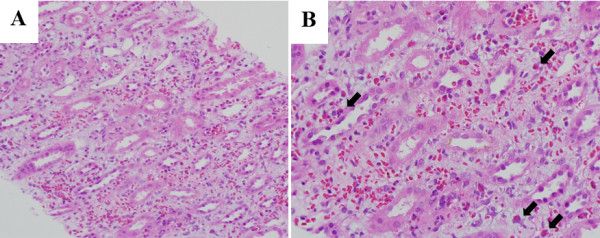
**The pathologic findings of the biopsy specimen were compatible with those of acute interstitial nephritis. A** Interstitial inflammation composed of eosinophils, lymphocytes, and neutrophils (×200, H&E stain). **B** Numerous eosinophils (arrow) in the interstitium and mild tubular cell flattening (×400, H&E stain).

A 52-year-old man was admitted with complaints of skin rash and burning sensation. He had noticed erythematous, variable-sized macules and patches on the dorsum of his feet and ankles (Figure 1), as well as swelling and redness on both metacarpophalangeal joints. He had ingested a raw extract of *D. quinqueloba* as a traditional remedy (Figure 2). The following day, he experienced symptoms of nausea, vomiting, and diarrhea. He had a history of hyperthyroidism and was treated with 5 mg of methimazole once daily. He denied the use of any other medication or supplements except for *D. quinqueloba*. On admission, the patient was afebrile, with normal blood pressure and pulse rate. Laboratory studies revealed the following values: white blood cell count, 9,900/mm^3^; absolute eosinophil count, 900/mm^3^; hemoglobin, 15.3 g/dL; platelet count, 193,000/mm^3^; aspartate aminotransferase, 27 U/L; alanine aminotransferase, 11 U/L; and total bilirubin, 0.65 mg/dL. Serological tests showed that the levels of anti-neutrophil cytoplasmic antibody, antinuclear antibody, complements, and double-stranded DNA were within the normal range. The urine output was 4.0 L/day. The serum creatinine level was 2.7 mg/dL, and blood urea nitrogen was 33.0 mg/dL. The level of immunoglobulin (Ig) E was markedly increased at 1320.0 IU/mL (institution laboratory reference of <100 IU/mL). Urinalysis showed a sodium concentration of 50 meq/L, fractional excretion of sodium of 3.77%, protein 1+, and blood 3+. The 24-hour urinary protein excretion was 930 mg/day. The kidney ultrasonography revealed slightly large and hyper-echogenic but morphologically normal kidneys, no scarring, good cortical preservation, and no evidence of hydronephrosis. On day 3, biopsies of the skin (Figure 1) and kidney (Figure 3) were performed. The kidney specimen contained 26 glomeruli, with no evidence of global or segmental sclerotic lesions. The glomerular basement membranes appeared normal, and there was no glomerular proliferation, inflammation, or other abnormality. Most of the changes involved the renal tubules and interstitium. Moreover, the interstitium was diffusely expanded by edema, with infiltrates composed of eosinophils, lymphocytes, and neutrophils. A limited number of red blood cell (RBC) casts were observed in the renal tubules and RBC extravasation was observed in the interstitium. Immunofluorescence staining was negative for IgA, IgM IgG, C3, C4, as well as kappa and lambda chains. Electron microscopy revealed no evidence of foot process effacement or electron-dense deposits. The pathologic findings of the kidney biopsy were compatible with those of AIN. The renal function progressively improved, and the patient was discharged with a serum creatinine of 1.1 mg/dL.

## Conclusions

*Dioscorea* is a genus of over 600 species of flowering plants in the monocotyledonous family *Dioscoreaceae*, mainly distributed throughout sub-tropical regions. *D. quinqueloba* is used widely in oriental countries as a traditional remedy [6], mainly for arthrosclerosis, myocardial infarction, and asthma.

Although it is difficult to diagnose AKI associated with the use of herbal medicines, the diagnosis can be strongly suspected on the basis of the diachronic relationship between kidney injury and ingestion of herbal medicines or an improvement in kidney function after discontinuation of the herbal medicines.

A review of the English medical literature revealed 3 previously reported cases with a clinical presentation suggesting AKI induced by the use of the same herbal medicines, one of which was the present case (Table 1) [5]. All patients were men and were aged 68, 58, and 52 years. They developed AKI, which was associated with an increased fractional excretion of sodium of >3%. All patients discontinued using the causative drug and were treated conservatively with saline hydration. The patient in this case also stopped taking *D. quinqueloba*, but he continued to take methimazole (5 mg). Renal function recovered without any steroid or immunosuppressant therapy. Renal function has been reported to recover in cases of AIN caused by many kinds of antimicrobial agents, non-steroidal anti-inflammatory drugs, analgesics, anticonvulsants, and diuretics [7]. Thus, the potentially causative agent should be immediately discontinued and conservative treatment should be initiated.

**Table 1:** **Summary of reported cases of acute interstitial nephritis induced by *Dioscorea quinqueloba***

**Case no.**	**Author**	**Age (years)/Sex**	**Underlying disease**	**Symptoms**	**BUN/Cr at admission**	**FeNa (%)**	**Urinalysis**	**Renal biopsy**	**Treatment**	**Length of hospital stay**	**BUN/Cr at discharge**
1	CS Kim et al.	68/M	Hypertension, Diabetes	Generalized edema, oliguria, vomiting, diarrhea	56.6/4.6	3.5%	Normal urinalysis results	N/A	Cease drug, hydration	53 days	15.3/1.2
2	CS Kim et al.	58/M	None	Pitting edema in the lower extremities, vomiting, diarrhea	48.2/5.8	6.4%	Proteinuria, hematuria	N/A	Cease drug, hydration	11 days	11.6/1.3
3	Present case	52/M	Hyperthyroidism	Skin rash, vomiting, diarrhea	33.0/2.7	3.7%	Proteinuria, hematuria	AIN	Cease drug, hydration	11 days	14.0/1.1

M, male; BUN, blood urea nitrogen (mg/dL); Cr, creatinine (mg/dL); FeNa, fractional excretion of sodium; N/A, not available; AIN, acute interstitial nephritis.

The renal biopsy findings in the present case are typical of drug-induced interstitial nephritis [7], with primary mononuclear inflammatory cell infiltrates observed mainly near the corticomedullary junction and clusters of eosinophils within the infiltrates. Immunofluorescent examination of the biopsy specimen revealed no immune deposits. After discontinuing the herbal therapy consisting of a raw extract of *D. quinqueloba*, the patient’s renal function progressively improved. To the best of our knowledge, this is the first case of biopsy-proven AIN induced by *D. quinqueloba*.

Immunological factors are believed to be important in the pathogenesis of drug-induced AIN. Classically, patients with drug-induced AIN were reported to have symptoms and/or signs of an allergic reaction, including rash, fever, and eosinophilia. However, a more recent review of 3 studies with a total of 128 AIN patients (of whom 70% had drug-induced disease) reported that a typical allergic response was relatively less common at presentation [8]. The findings in the present case, including skin rash, eosinophilia, and elevated Ig E level, may provide a clue in the diagnosis of AKI due to *D. quinqueloba*. The representative chemical composition of the *Dioscorea* species is dioscorine and dioscine, which is known to cause allergic reactions [9]. In previous reports, immunoblotting with an extract of *Dioscorea batatas* showed increased levels of plasma IgE and IgG4 antibodies to *D. batatas*[10].

In summary, we presented a case of biopsy-proven AIN, in which *D. quinqueloba* was the only obvious precipitating factor for the histologic changes and impaired renal function. Therefore, we believe that *D. quinqueloba* contributed to the pathogenesis of AIN. The patient experienced a typical allergic reaction, characterized by skin rash, eosinophilia, and elevated IgE level. In addition, the renal pathologic findings revealed AIN, characterized by infiltration of many inflammatory cells in the renal interstitium. Overall, we recommend obtaining a thorough medical history including herbal medicine intake to avoid a delay in the diagnosis of AKI. Moreover, a detailed investigation including renal biopsy should be performed, as a histopathologic diagnosis is critical in the management of drug-induced AKI.

## Consent

Written informed consent was obtained from the patient for the publication of the present case report and any accompanying images.

## Abbreviations

AIN: Acute interstitial nephritis; AKI: Acute kidney injury; Ig: Immunoglobulin; RBC: Red blood cell.

## Competing interests

The authors declare that they have no competing interests.

## Authors’ contributions

HYK carried out the final preparation of the manuscript. SSK and SHB participated in the histological review of the biopsies. EHB and SKM contributed to the discussion in the conclusion, and critically reviewed the manuscript. SWK contributed to the final preparation of the manuscript. All authors contributed to the content and approved the final version of the manuscript to be published.

## Pre-publication history

The pre-publication history for this paper can be accessed here:

http://www.biomedcentral.com/1471-2369/15/143/prepub
